# Changes in the EV-A71 Genome through Recombination and Spontaneous Mutations: Impact on Virulence

**DOI:** 10.3390/v10060320

**Published:** 2018-06-12

**Authors:** Madiiha Bibi Mandary, Chit Laa Poh

**Affiliations:** Centre for Virus and Vaccine Research, School of Science and Technology, Sunway University, Kuala Lumpur, Selangor 47500, Malaysia; madi.mandary@live.com

**Keywords:** EV-A71, epidemiology, recombination, quasispecies, spontaneous mutations, virulence

## Abstract

Enterovirus 71 (EV-A71) is a major etiological agent of hand, foot and mouth disease (HFMD) that mainly affects young children less than five years old. The onset of severe HFMD is due to neurological complications bringing about acute flaccid paralysis and pulmonary oedema. In this review, we address how genetic events such as recombination and spontaneous mutations could change the genomic organization of EV-A71, leading to an impact on viral virulence. An understanding of the recombination mechanism of the poliovirus and non-polio enteroviruses will provide further evidence of the emergence of novel strains responsible for fatal HFMD outbreaks. We aim to see if the virulence of EV-A71 is contributed solely by the presence of fatal strains or is due to the co-operation of quasispecies within a viral population. The phenomenon of quasispecies within the poliovirus is discussed to reflect viral fitness, virulence and its implications for EV-A71. Ultimately, this review gives an insight into the evolution patterns of EV-A71 by looking into its recombination history and how spontaneous mutations would affect its virulence.

## 1. Introduction

Enterovirus 71 (EV-A71) belongs to the genus *Enterovirus* in the family *Picornaviridae* and is classified as a human enterovirus species A (HEV-A) based on its genome sequence. Phylogenetic analysis of the VP1 region of EV-A71 has shown that the virus can be grouped into three main genotypes: A, B and C. Genotype A is represented by the prototype BrCr strain whereas genotypes B and C can be further segregated into five subgenotypes, i.e., B1 to B5 and C1 to C5 respectively [1]. Newly discovered isolates were classified as genotypes D (isolated from India), E and F (isolated from Africa) [2].

EV-A71 was first isolated and characterized from a child with severe neurological disease from California in 1969 and since then, many hand, foot and mouth disease (HFMD) outbreaks have been reported around the world [3]. EV-A71 together with CV-A16 (Coxsackievirus-16), CV-A6 and CV-A8 are the main causative agents of HFMD and these pathogens have caused millions of infections in China and South East Asian countries from 2008 to 2016. In 2015, a total of 2,014,999 cases of HFMD including 124 deaths were reported in China. Less than 12 months later in August 2016, 1,792,251 cases of HFMD were recorded by WHO, including 172 fatal cases in China [4]. Complications of HFMD infections include aseptic meningitis, brainstem encephalitis, poliovirus-like paralysis, shock and cardiac dysfunction [5,6].

Early reports of severe HFMD occurring in countries such as Bulgaria [7] and Hungary [8] have been documented since 1975 to the early 80’s. However, the last decade has witnessed a significant increase in epidemic activities of EV-A71 in Asia. Countries in the Asia Pacific region such as Malaysia, Taiwan, Singapore, Vietnam and China have witnessed recurrent outbreaks of EV-A71 epidemics since the late 1990’s. A large number of HFMD-associated fatal cases were recorded in Malaysia, Taiwan and China. In April to June 1997, 2628 EV-A71 cases were recorded in Sarawak, East Malaysia [9]. During this outbreak, 889 children were hospitalized and 39 developed aseptic meningitis, leading to acute flaccid paralysis. A total of 29 children younger than six years of age died of rapidly progressive cardiorespiratory failure. In Taiwan in 1998, 78 fatal cases of HFMD amongst 100,000 hospitalized patients were reported during a severe EV-A71 outbreak. Another large outbreak occurred in Taiwan again in 2000, with 41 deaths being reported amongst 80,677 HFMD patients [10]. In China, enteroviruses such as EV-A71, CV-A16 and other enteroviruses have caused 7,200,092 HFMD cases from 2008 to 2012 and the mortality was highest among children aged 1 to 3 years of age. It was further reported that 82,486 patients developed neurological complications, and 1617 deaths were confirmed by the laboratory to be caused by EV-A71 [11].

**Figure 1:**
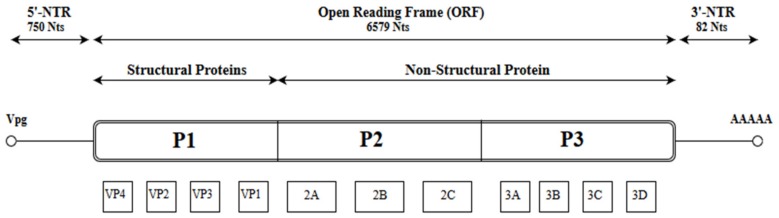
Schematic representation of the EV-A71 genome (7.4 Kb). The position of the VPg primer is shown at the 5′ NTR end of the genome; the Open Reading Frame (ORF) contains the structural viral protein P1 which is cleaved to yield VP1, VP2, VP3 and VP4, and non-structural viral proteins P2 (cleaved to yield 2A, 2B and 2C) and P3 (cleaved to yield 3A, 3B, 3C and 3D). The 3′ NTR end of the genome contains the poly (A) tail.

Like other picornaviruses, EV-A71 contains single-stranded positive RNA (+ssRNA) which is contained within the non-enveloped capsid of the virus [12]. The RNA genomic sequence is made up of 7411 base pairs and has an open reading frame (ORF) flanked on both ends by the 5′ Non-Translated Region (5′-NTR) and the 3′ Non-Translated Region (3′-NTR) [13]. The 5-NTR has an internal ribosome entry site (IRES) which controls cap-independent translation. The ORF comprising 6579 nucleotides can be classified into three polyprotein regions, namely, P1, P2 and P3. They encode for structural proteins (VP1 to VP4) in the P1 region and non-structural proteins in the P2 (2A-2C) and P3 regions (3A-3D) following proteolytic cleavage (Figure 1) [5]. The viral capsid proteins VP1, VP2 and VP3 are displayed on the external structures of the EV-A71 viral particle whereas VP4 is found within the internal structures of the capsid. The 2A and 3C proteins are of particular interest as they are crucial for viral-host interactions, viral replication and apoptosis. As for the 3D polymerase of a +ssRNA virus, it is an RNA-dependent RNA polymerase (RdRP) with low fidelity, having as many as 10^−3^ to 10^−5^ mutations per nucleotide copied in each replication cycle, and this could result in misincorporation of 1–2 new nucleotides. [14,15].

The rate of mutation in RNA genomes was reported to be in the order of 10^−4^ to 10^−6^ fold higher than the mutation rate of a stable DNA genome of similar complexity [16]. It was established that such high rates of mutation are due to the absence of proofreading-repair activity in the viral RdRp [17]. The interaction between mutation and recombination enables viruses to benefit from the advantages of having a few beneficial mutations without being affected by many deleterious mutations.

## 2. Recombination

The first case of RNA recombination was originally reported in poliovirus by Hirst (1962), and since then many studies have demonstrated that recombination is significant and frequently occurring in enteroviruses [18,19]. Since its discovery, recombination in RNA viruses has been abundantly documented and it was further revealed that recombination might occur within a specific serotype or between different serotypes of enteroviruses. The evolution and emergence of enteroviruses through intra- and inter-species recombination in the 5′ untranslated region have also been demonstrated [20]. In this review, we look into the different types of recombination between poliovirus and non-polioenteroviruses and how they affected the evolution of RNA viruses such as EV-A71.

### 2.1. Recombination between EV-A71 and Other Enteroviruses

The exchanges of genomic domains between poliovirus and other co-circulating enterovirus species affected the plasticity of enteroviral genome. Specifically, the extent of genetic exchanges that exist between circulating vaccine-derived polioviruses (cVDPV) and non-polioenteroviruses of species C (EV-Cs) that could affect functionality was investigated through construction of chimeric viruses [21]. The analysis revealed good genome functionality for most recombinant viruses and they replicated similarly in vitro but yielded different plaque sizes and temperature sensitivities. The study puts forth the fact that most complex recombinant genomes generated from the Sabin strain and nonpolio-EVs had a particular sequence arrangement: the EV sequences were mostly located within the P2, P3 and 3′-NTR of the genome and in many cases within the 5′-NTR. Data collected over the three-year period suggested that the preferred position of the EV sequences within the recombinant mosaic genome was due to the replication mechanism favouring exchanges by intertypic recombination and functional compatibility of the domains exchanged. Interestingly, recombinant viruses differed in terms of their plaque sizes when recombination events occurred within the 5′-NTR or P2, P3 and 3′-NTR. For instance, 17 out of 38 recombinant viruses generated larger plaques than Sabin 2 strains while a few (3/28) generated smaller plaques. These highly diversified plaque phenotypes showed the significant impact of recombination on the mechanism of an underlying genotype. It is worthwhile to note that of those recombinant viruses exhibiting different plaque phenotypes, the best adapted to survive is likely the candidate that could spread rapidly within a population. Moreover, substitutions within the P2 and the P3/3′-NTR region of the Sabin 2 genome were not seen to confer a neurovirulent phenotype on the recombinant viruses and they exhibited attenuated phenotypes. In contrast, recombination of the Sabin 2 genome with the 5′-NTR of the non-polio EV-Cs was observed to generate a virulent recombinant which induced paralysis of 25–50% in mice, therefore indicating an increase in the virulence of the Sabin 2 recombinant viruses [21].

It is intriguing that most of the outbreaks documented in the Asia-Pacific region in the last few decades arose from previously circulating genotypes/subgenotypes of EV-A71 and this raises a question concerning their origin and genetic complexity [22]. A ladder-like tree structure was observed for B1 to B4, C1, C2 and C3 subgenotypes of EV-A71 and the patterns of the phylogenetic structure put forward the idea that a temporal strain replacement occurred through time on a global scale. This suggestion stems from the fact that viral sequences isolated at early time points are located closer to the tree root while sequences collected at a later time point are found further away from the tree root together with limited genetic diversity observed at any one time. However, homologous recombination in the VP1 gene was detected less frequently than recombination outside the VP1 region as functional constraints tend to limit recombination in the capsid genes [23]. It is important to note that recent outbreaks of HFMD in different countries caused by the same EV-A71 subgenotype might have arisen from different lineages rather than through the sequential transmission of a single lineage [24].

**Figure 2:**
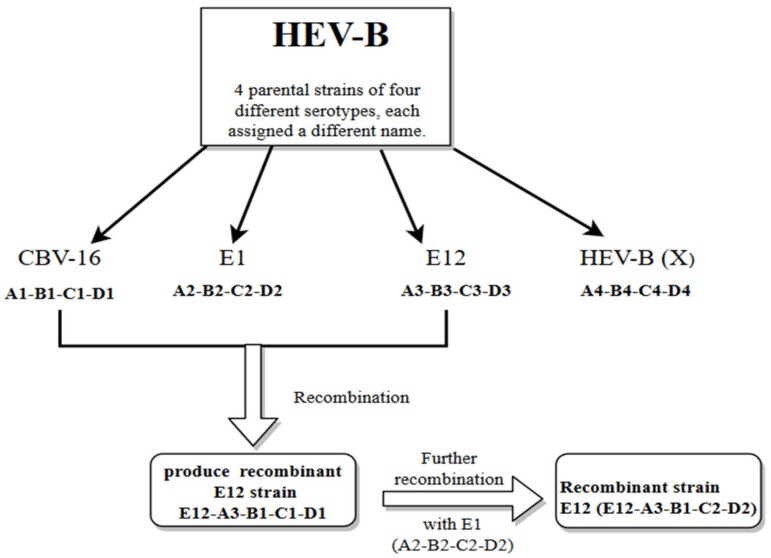
Illustration showing the two events of recombination of HEV-B (CBV16, E1, E12 and X) using similarity plots. CBV-16, E12, E1 and X refer to the designated names of four ancestral strains of four different serotypes. The white arrows indicate the recombination events between the two ancestral strains (CBV-16 and E12) and further recombination with E1.

Further evidence for recombination in enteroviruses was demonstrated by looking into the pattern of similarities between the P2 and P3 regions of Human Enterovirus-B (HEV-B) using similarity plots. These data showed high similarity ranges from a relatively low (60–80%) to a relatively high degree of similarity (80% to 95%) between many strains throughout most of their P2 and P3 regions. In some cases, it is possible to rebuild the recombination history of some clinical isolates to infer the minimum set of recombination events that were needed to produce the set of isolates. This was shown by analyzing the relationships among four different serotypes of HEV-B, each assigned a designated number: CBV16, E1, E12 and X based on previous studies [25]. These parental strains were considered to hold four distinct regions (A-D) and within each distinct area of the genome, an allele number was allocated (1–4) to ease the identification process of the recombined segments. Interestingly, recombination of CBV-A1-B1-C1-D1 with E12-A3-B3-C3-D3 between regions A and B was observed to produce strain E12-A3-B1-C1-D1 which denotes the association of E12 capsid with the B1 allele of CBV-16. Further recombination analysis revealed that recombination events between the recombinant E12-A3-B1-C1-D1 with the E1 serotype (E1-A2-B2-C2-D2) occurring between the regions B and C produced a further recombinant E12 (E12-A3-B1-C2-D2) and the E12 capsid was associated with the C2 allele. Hence, this indicated that the new isolates carried evidence of recombination (Figure 2) [25].

It is important to highlight that EV-A71, as a RNA virus, could undergo copy-choice recombination in order to generate intertypic and intratypic recombinant EV-A71 viruses. Copy choice recombination could occur when the RNA-dependent RNA polymerase dissociated from the genome of a virus and continued to replicate by binding to a second genome, which then generated a new mosaic-like genome with regions originating from different parental strains [26,27]. It was demonstrated that a mutation (D79H) in the RdRp of PV was able to reduce the rate of recombination without changing the fidelity of replication. By creating a range of variants with specific mutation rates and recombination ability, the authors revealed that recombination is crucial to build up beneficial mutations and suppress deleterious mutations. Since it was established in the case of poliovirus that homologous recombination is mediated by template switching during replication (copy-choice recombination) [28], it could be deduced that other enteroviruses (such as EV-A71) would also follow the same mechanism of recombination (Table 1). This deduction follows the rationale that EV-A71 has a (+) ssRNA much like poliovirus.

It is apparent that recombination events are not conserved to a particular gene or set of genes of the EV-A71 but rather seem to span across the entire length of the genome [24,29,30,31,32,33] (Table 1). Other supporting studies on the evolutionary rate and the time of emergence of different subgenotypes of EV-A71 revealed that the evolutionary rate of the VP1 gene of EV-A71 was approximately 4.6 × 10^−3^ and 4.2 × 10^−3^ substitutions/site/year for genotypes B and C, respectively. The common ancestors of subgenotype B1 (identified in 1972) have been circulating since 1967 before EV-A71 was classified as a human pathogen. Since then, other subgenotypes such as B2 (in the late 1970’s), B3, B4 (in the mid 1990’s) and B5 (early 2000) were discovered. By comparing the estimates of origin and the date of first detection of each subgenotype, it could be implied that different subgenotypes of EV-A71 genotype B have been circulating for approximately 2 to 5 years before a recombinant virus emerged to cause a large HFMD outbreak [22]. The genetic diversity and population dynamics addressed whether a rise in EV-A71 cases in China reflected a real increase in the viral spread of a major sub-genotype or it was due to different sub-genotypes. Analysis of 257 EV-A71 subgenotype C4 strains from China between 1998 to 2010 revealed an increase in viral spread and continuous replacement of viral lineages by mutations in VP1 through time [33].

Understanding the recombination processes existing between non-polioviruses is very important. It can give an insight into the evolutionary pattern of EV-A71 by looking into its recombination history throughout the years and might lead to the prediction of new patterns of recombination. Analysis of the EV-A71 genome showed that recombination events between EV-A71 and Coxsackieviruses are key factors in the evolution of EV-A71 genotypes/subgenotypes (Table 1). Furthermore, investigating the intertypic and intratypic relationship is an opportunity to discover any possible epidemic strain that may arise to cause large outbreaks in the future. Genome similarities between coxsackieviruses and EV-A71 reported in this review are core to the idea whereby it can be observed that new recombinants could arise from recombination of part of the EV-A71 genome with coxsackieviruses such as CV-A16, CV-A5, CV-A8, CV-A10 and CV-A12. The new recombinants may have acquired virulence determinants to cause major fatal outbreaks as observed in China in recent years.

### 2.2. Recombination of Poliovirus and Its Implication for EV-A71

**Figure 3:**
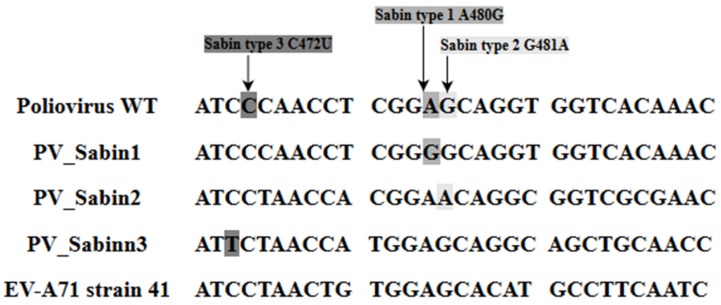
Comparison of the nucleotide sequence between the Wild Type Poliovirus, Poliovirus Sabin Strain 1, 2 and 3 and EV-A71 strain 41(GenBank: AF316321). PV Sabin 1, PV Sabin 2 and PV Sabin 3 contain altered nucleotides in the 5′-NTR of ssRNA. Shaded areas in different shades of grey indicate the location where the altered nucleotides are.

Poliovirus was nearly eradicated due to worldwide immunization efforts. With the absence of effective vaccines against EV-A71, it may become the next important neurotropic pathogen to replace poliovirus and pose a significant health threat to humans [30]. There are some marked similarities between poliovirus and EV-A71. The complete nucleotide and polyprotein sequences of EV-A71 reflect the normal enterovirus organization and composition. This similarity is postulated to be the reason why EV-A71 is able to give rise to clinical complications that resemble poliomyelitis and neurological complications such as acute flaccid paralysis [13]. Even though low scores of nucleotide similarity (58%) between poliovirus and EV-A71 indicate that they are not genetically highly related overall, it is worthwhile noting that the significant neurovirulence determinants present in the 5′-NTR of the wild type Poliovirus are similar to some nucleotides in the 5′-NTR of EV-A71 subgenotype B4 strain 41 (GenBank: AF316321) (Figure 3) [13].

This observation was supported by the hypothesis that EV-A71 could have evolved from recombination between part of the genome of CV-A16 and 5′-NTR of poliovirus. It was revealed that the nucleotide identity of the EV-A71 (EV71/MS/7423) strain with the CV-A16 and EV-A71 prototype BrCr strains is high, at 77% to 81%. However, the overall nucleotide identity to Poliovirus is only 58%. The amino acid identity of the EV71/MS/7423 to both EV-A71 BrCr strain and CV-A16 is high, at 95% and 89%, respectively. But, the overall amino acid identity to poliovirus is only 55% (Table 2) [13]. The greater identity between EV-A71 and CV-A16 can be seen as consistent given the fact that both EV-A71 and CV-A16 are the etiological agents of hand, foot and mouth disease [34].

Some studies have aimed to uncover the evolutionary strategies by which vaccine strains of poliovirus have reverted back to their virulent genotypes, thus providing an insight for forecasting the virulence of viruses and the framework for rational design for vaccine strains. Since recombination events were recorded in the P2 and P3 regions of a circulating vaccine-derived poliovirus type 2 (cVDPV2), further understanding of the evolutionary changes contributing to the regain of virulence from attenuation was desired. To this end, further experiments showed that the recombination with HEV-C strains consistently contributed to changes in the 3C gene, the 3′-NTR and the 3D gene of the OPV2 genome. This would explain why most sequences of the cVDPV2 were recombinant in certain specific regions. Since the donating sequence (HEV-C) supplied the mutations together with transition mutations already present in the OPV2 genome, the selection of certain mutations in the recombinants could be due to epistatic interactions with human cellular components [35]. In light of these results and considering the startling similarity between the poliovirus and EV-A71 genome, it would be pertinent to question whether epistatic interactions could be a driving force influencing recombination events and mutations in EV-A71 strains, leading to production of different strains causing large outbreaks every year in China.

Another façade of the mechanism of copy-choice recombination that is crucial for viable and competent recombinant RNA viruses to arise consists of the numerous constraints from the host and co-infection. For instance, a prerequisite for a fruitful recombination event is co-infection of the host by different virus strains. This step can be thwarted by the immune response of the host that keeps the number of viruses low or genetic factors of the cell that stopped multiple entry of different viruses into the target cell [36]. Upon successful co-infection, the second criterion towards copy-choice recombination is that both RNA viruses should undergo replication at the same time. This enables the RdRp to switch template and create recombinant RNA viruses. However, the likelihood of divergent viruses replicating in the presence of one another was reported to be extremely rare and not necessarily inevitable within the co-infected cell. Finally, the ability of the viral polymerase to switch from one template to another remains the ultimate determining factor on how often the viruses will recombine. After overcoming the virus or host constraints, whether the nascent recombinant viruses become viable recombinant hybrids depends on whether they survive selection. Through passages, selection provides the opportunity for less fit variants to be removed [27]. This gives a better insight into the mechanism by which EV-A71 and a diverging RNA virus such as CV-A16 or an EV-A71 of a different genotype which might have co-infected a cell in order to generate recombinant viruses that are able to pass the positive selection and emerge to circulate in a population. Therefore, by dissecting the process which gives rise to recombinant viruses and considering the different restraints that occur to inhibit it, the evolutionary advantages conferred by recombination such as the removal of deleterious mutations and the spreading of advantageous traits [27,28] could be understood. Furthermore, the availability of a mutation that decreases the rate of recombination but does not directly change the viral fitness would give an unprecedented opportunity to explore the interplay between mutation and recombination in driving adaptation of EV-A71. In turn, this will facilitate an understanding of constructing a stable and attenuated strain which makes an ideal vaccine candidate.

Another important aspect which plays a role in driving the virulence of EV-A71 could be the fidelity of the enteroviral polymerase and its interaction with host molecules. In an attempt to understand the role of the 3D polymerase in the virulence of EV-A71, it was discovered that the 3D^pol^ entered the nucleus and targeted the pre-mRNA processing factor, prp8 to block pre-mRNA splicing and mRNA synthesis of the cell. Specifically, the finger domain of the 3D^pol^ was found to associate with the C-terminal region of the prp8 which contained the Jab1/MPN domain. This interaction thus interfered with the second catalytic step, inhibiting mRNA production and inducing the build-up of its lariat form in the nucleus. This novel mechanism of viral attack contributed to the pathogenesis of the EV-A71 viral infection. However, this novel mechanism was only observed in certain viruses such as EV-A71 and PV. Other viruses such as CVB-3 and HRV-16 did not inhibit pre-mRNA splicing as it could not associate with the C-terminal of the cellular prp8 [37]. Since recombination is a fairly common occurrence in EV-A71, this particular study raises the question whether recombination between EV-A71 and another enterovirus such as coxsackievirus would affect the virulence of the newly evolved recombinant virus. Following copy-choice mechanism of recombination, the recombinant virus would possess a 3D^pol^ which lacked the ability to stop pre-mRNA splicing. This would ultimately affect the virulence of the recombinant virus whereby it would have restricted access to the host machinery to make more viral RNA [37].

It was investigated whether mutations with the ability to decrease recombination affected the fidelity of replication. The mutation D79H was observed to have reduced recombination but no impact on fidelity, and recombinant viruses derived from the D79H mutant displayed a reduction in the accumulation of advantageous alleles and an increase in detrimental mutations. Using CirSeq analysis, the frequency of beneficial mutations was found to increase 100-fold over 7 passages for the wild type virus. However, no accumulation of beneficial mutations was observed for single mutants D79H and H273R. It is important to note that combining the mutations affecting fidelity and recombination resulted in major defects in the accumulation of beneficial mutations [28]. More in-depth analysis aimed to understand whether double mutations affecting recombination and fidelity inhibited viral adaptation in animals. Interestingly, the wild type virus was observed to cause fatality within 6 days while the recombination-deficient strain carrying the mutations D79H was as lethal as the WT. As for the low-fidelity mutant H273R, a milder phenotype was reported. Combining either high-fidelity or low-fidelity mutation with recombination-impairing mutations would dramatically reduce the virulence. It would seem that decreasing the rate of recombination as well as changing the replication fidelity could lower the ability of the virus to adapt within the infected host, resulting in a strong attenuation phenotype [28]. From the lethal dose (LD_50_) observed, the lack of viral adaptation led to the alteration in virus tissue tropism which stopped the onset of infection in the CNS while allowing wild type levels of replication in the non-neuronal tissues [28].

Akin to these findings, it would be interesting to explore whether the RNA polymerase in EV-A71 could bear mutations which would decrease the recombination rate and affect viral fitness and adaptation. Findings from such an experiment would help to shed some light on and track the successive emergence and disappearance of EV-A71 lineages. Ultimately, a clearer picture on the evolution strategies of EV-A71 would have a greater bearing on its vaccine design.

## 3. Spontaneous Mutations

### 3.1. Spontaneous Mutations in the 5′-NTR of EV-A71

When the nucleotide cytosine at position 158 was substituted with uridine, the conformation of the RNA secondary structure of stem loop II within the 5′-NTR was changed, leading to a decrease in viral translation and increased virulence in mice [38]. Further evidence of spontaneous mutations affecting virulence of C4 and C4a subgenotypes was reported at three different positions: Val814/Ile814 in the VP1, ValP1148/IleP1148 in the 3A and AlaP1728/CysP1728 and ValP1728 in the 3C genes. These amino acids were highly conserved in the neuro-virulent strains of the subgenotype C4a. The implication from this study is that these amino acids are potential molecular determinants of virulence of EV-A71 subgenotype C4a. Variations of the secondary structure of the 5′-NTR were also observed in the fatal strain at three positions (CP241/TP241, AP571/TP571 and CP579/TP579) and one position in the 3′-NTR (TP7335/CP7335) and these amino acids might confer fatality [39]. When the sequences of EV-A71 responsible for severe and mild HFMD cases (25 SC-EV-A71 and 31 MC-EV-A71) across different genotypes (A, B1-B5 and C1-C4) were compared, they showed that 4 amino acid residues (GlyP710/GlnP710/ArgP710/GluP729) at 2 positions in the VP1 (VP1-145G/Q/R and VP1-164E), one residue (LysP930) in protease 2A and four nucleotides at three positions (GP272, UP488 and AP700/UP700) in the 5′-NTR might be associated with the EV-A71 virulent phenotype [40].

### 3.2. Spontaneous Mutations in the VP1 of EV-A71

Nine amino acid substitutions at different positions in the EV-A71 genome, namely: H22Q, P27S, N31S/D, E98K, E145G/Q, D164E, T240A/S, V249I, and A289T obtained from the alignment of VP1 sequences of the SC-EV-A71 (strains causing severe HFMD) versus the MC-EV-A71 (strains causing mild HFMD) were reported to have arisen spontaneously. The changes in these amino acids might play a role in conferring virulence to the EV-A71 subgenotype C4 strain [41]. The authors hypothesized from these data that the VP1 of the EV-A71 genome could act as a sandwich switch for the stabilization of viral particles and cellular receptor attachments. When EV-A71 carries VP1-145G/Q, it has the ability to interact with the residues of the cellular receptor, PSGL-1 N-terminus and as a molecular switch to modulate viral binding to the cell receptor by controlling the exposure of the amino acid (VP1-244K) on the VP1 surface [42]. Moreover, the critical substitution of E-Q at amino acid position 145 within the VP1 occurred multiple times in different virulent strains responsible for severe cases of HFMD [43]. Therefore, specific mutations such as the E145G/Q detected in the VP1 of SC-EV-A71 have the ability to convert EV-A71 strains associated with mild infections to strains capable of causing severe HFMD [41]. Other spontaneous mutations such as K215A located at the VP1 GH loop were observed to increase the thermal stability of the virus, and thermostable mutants could be produced by changing the positively charged residues to alanine by alanine scanning of the VP1 [44].

It has been reported previously that an important contributor to virulence is a group of positively charged amino acids near the 5-fold vertices of the EV-A71 capsid, and many of these positively charged amino acids are conserved across EV-A71 genotypes, suggesting their importance to the virus. Identification of single mutations in the conserved, positively charged 5-fold region of the VP1 capsid protein might therefore affect virulence in a significant way. Caine et al. (2016) discovered a mutation at nt. 244 (K244E) in VP1 which is crucial for the virulence of the mouse-adapted EV-A71 (mEV-A71) strain as it led to the expansion of tissue tropism and viral spread in adult interferon-deficient AG129 mice. Another spontaneous VP1 mutation (H37R) arose when mEV-A71 was passaged in primate cells, and H37R was found to be important for the recovery of mEV-A71 in rhabdomyosarcoma cells (RD cells). It was postulated that H37E and K244E interactions are important for replication in primate cells but K244E alone was able to confer the ability of the virus to replicate alone in mice [45].

In a more recent study, molecular modelling indicated that mutations in the 5′-NTR and the VP1 influenced the EV-A71 engagement with human receptors SCARB2 and PSGL-1 and the virulence in vivo. During EV-A71 propagation in Vero cells and RD cells, mutations leading to nucleotide substitution (T494C) in the 5′-NTR and mutations E145G, V146I and S241L in the VP1 altered virulence such that propagation of EV-R in the RD cells exhibited lower virulence and pathology in mice. The authors speculated that mutations in the VP1 affected the structural conformation of the capsid which in turn altered the ability of the virus to bind to the cellular receptor. To investigate this speculation, interactions of the EV-V with SCARB2 were carried out and results indicated that EV-V propagated in Vero cells had more interactions with SCARB2 compared to EV-R propagated in RD cells. In contrast, EV-R propagated in RD cells had a higher affinity to PSGL-1. Hence, propagation in different host cells drives the virus to adapt to the physiological environment via mutations in the VP1 [46]. Consequently, when a virus undergoes mutations which lead to a change in the surface proteins, it is important to understand that antibodies elicited by an inactivated vaccine in previous vaccinations would not be effective to neutralize any mutated virus that might arise [47]. Thus, there is a real need to study any potential mutations which could compromise the efficacy of a vaccine.

### 3.3. Spontaneous Mutations in the 2A and 3C of EV-A71

Some spontaneous mutations such as the non-synonymous mutation (K129→I) within the ORF of a mouse-adapted strain of EV-A71 was established to be responsible for an increase in virulence in mice. Identification of the K216→R within the 2C protein demonstrated an improved growth of the strain in vitro but did not lead to increased virulence in mice [48]. A critical mutation characterized by Asn1617 within the 3C protease gene was present in highly virulent strains which was different from Asp1617 present in strains with low virulence. This highlights the position of this specific amino acid which led to a conformational change at the active center of the 3C proteinase (3Cpro) and could serve as a potential molecular determinant of virulence in the EV-A71 subgenotype C4a [49]. These findings establish the significance of single site mutations which arose spontaneously within the viral genome and have an impact on the growth in vitro and in vivo (Table 3).

When the strains of EV-A71 subgenotype C4, isolated from severe HFMD, were compared to those isolated from mild HFMD, the majority of mutations were located in the 5′-NTR and the VP1 regions (Table 3). For instance, 10 mutations were observed in the VP1 alone [38,41,42,44]. However, when 2 strains of EV-A71 subgenotype C4a [39,40] were compared with the C4 subgenotype, the genes affected by the spontaneous mutations appeared to span across the entire length of the genome, ranging from the 5′-NTR to the 3′-NTR. It would be compelling to investigate the virulence of these strains by quantifying the degree of virulence conferred by each of the mutated genes so as to gain a better understanding on how exactly they affect virulence.

**Figure 4:**
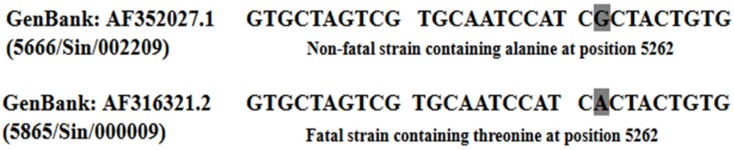
Comparison of a fatal (AF316321.2) and a non-fatal (AF 352027.1) strain isolated from the HFMD outbreak in Singapore (2000). Highlighted in grey shows the difference of a single nucleotide (A) was observed at nucleotide position 5262 (amino acid residue 1506) in the 3A non-structural region of the fatal strain [50].

Interestingly, when the fatal EV-A71 strain isolated from the severe HFMD outbreak in Singapore in 2000 was analyzed, it was found to differ from the non-fatal strain C10 (5666/Sin/002209) only at the nucleotide position 5262. The fatal strain carried an A nucleotide at position 5262 of the genome (Figure 4) and this led to the replacement of alanine in the non-fatal strain with threonine in the fatal strain [50]. To check whether threonine at this position could have contributed to the fatality of the strain, Yee et al. (2016) quantified the virulence of the fatal strain (C41) versus the non-fatal strain (C10) by site directed mutagenesis of the A→G nucleotide. The mutation at position 5262 (A5262G) led to a 75% reduction in viral RNA copy number and 90% reduction in plaque forming ability in the non-fatal strain when compared to the fatal strain [51].

Yee et al. (2016) further evaluated the effects of specific mutations of the EV-A71 subgenotype B4 strain 41 genome on virulence by mutating at multiple positions (158, 475, 486 and 487) and through partial deletion of 11 base pairs from nucleotides 475 to 486 in the 5′-NTR region. This strain was shown to encode for several significant molecular determinants. The mutant 475 (C475T) and the partial deletant (PD) (Δ 11bps, from 475 to 486 in the 5′-NTR) showed significantly lower cytopathic effects and very low viral RNA copy numbers. However, analysis of RNA copy number showed that mutant A486G still produced high levels of viral RNA in the RD cells while mutant G487A showed minimal levels of RNA copy numbers in the RD cells. The data provided quantitative comparisons of the molecular determinants of virulence in the EV-A71 subgenotype B4 strain 41 [51].

Published data have shown that there were amino acid differences between fatal and non-fatal strains isolated from China [40,41,43,49]. Does this imply that some strains with higher virulence are able to cause large-scale outbreaks? From the data presented, both intratypic and intertypic recombination could generate such a highly virulent strain. The strong likelihood that multiple errors arise each time the EV-A71 virus replicates also raises the question as to whether a single mutation such as that observed in the VP1-145 is really the root cause of fatality or rather, a community of mutants carrying multiple amino acid substitutions increases the viral fitness of the viral population, thereby conferring higher pathogenicity. Under normal circumstances, a viral genome replicates and creates hundreds of progeny viruses which could differ by an amino acid substitution at one position. In the following rounds of replication, more complex mutants could be generated which could differ significantly from the original master sequence and this ensemble of mutants form a quasispecies [52]. It would be pertinent to question whether a single EV-A71 fatal strain arising from recombination and/or spontaneous mutations is the only rationale behind sudden outbreaks of HFMD in various countries or there could be the cooperation of diverse quasispecies with varying virulence. Whether this claim stands true remains to be confirmed.

## 4. The Phenomenon of Quasispecies

Quasispecies have been well documented for viruses such as poliovirus and rabies virus. Population diversity may have a role in poliovirus pathogenesis, specifically in the ability of the virus to spread to the central nervous system (CNS). Although the wild type virus could access and replicate in the CNS, a high fidelity virus would be restricted from systemic spread. Following treatment of the high fidelity virus with mutagenic nucleoside analogues, genomic diversity was restored and it was able to spread to the CNS.

In an attempt to look into the implication of quasispecies of rabies virus on pathogenesis, the discovery of the existence of subpopulations within the CVS-24 strain of rabies virus that had implications on its virulence was discovered. More specifically, the discovery of differences in the pathogenicity of the two chosen strains (CVS-B2c and CVS-N2c) was observed. When the relationship between these two strains was studied, the pathogenesis index divided by the viral titre was higher for the CVS-B2c strain than for the CVS-N2c strain in the neonatal mice. The CVS-B2c strain was detected in infected muscle tissue 24 h after infection but not for the CVS-N2c until 48 h later. This implied that only the CVS-B2c strain was able to replicate at the site of infection, and the early appearance of rabies antigen in the periphery could stimulate an early response to that particular subpopulation of the viral strain. This implication was further confirmed by a more rapid development of neutralizing antibody for the CVS-N2c strain than the CVS-B2c strain. It was postulated that the two existing variant strains within the original rabies virus population overcame barriers to facilitate its spread within the host and between species [53].

Pfeiffer et al. (2006) demonstrated that four restriction site tagged polioviruses of equal viral fitness were able to replicate in the mouse brain. However, when the infections using the same polioviruses were initiated using different routes (intraperitoneal, intramuscular and intravenous), only a subset of peripherally inoculated polioviruses was detected in the brain, even though each of the viruses was initially capable of replicating and spreading to the brain. The authors argue that a bottleneck effect could have affected the viral transit from the inoculation site to the brain. This bottleneck effect was difficult to overcome and required 10^7^ more viral inocula to allow representation of most members of the viral population in the murine brain. Hence, this strong bottleneck effect is likely not a physical barrier but an antiviral state induced by the ‘founder’ virus. It was suggested that the host innate immune response could limit viral pathogenicity by limiting the number of viruses and consequently viral diversity during infection [54].

Very recently, the discovery of the evolutionary pathway by which EV-A71 evolved fitness to invade the central nervous system in humans was observed. This was achieved using deep sequencing and haplotype analysis of viruses isolated from different tissues of the digestive, integumentary and central nervous system. Data collected identified a new selection bottleneck appearing in the respiratory system, digestive system and the CNS during EV-A71 dissemination in humans. This bottleneck effect drove a dominant haplotype switch from viruses having VP1-31D to VP1-31G whereby VP1 residue 31 was a common positive-selection site among circulating EV-A71 subgenotype C2. The VP1-31G viruses were also detected in higher proportions among the mutant spectra of EV-A71 in the throat swabs of patients with fatal EV-A71 infections. In vitro experiments indicated that VP1-31D variants had increased virion stability, infectivity and fitness which maintained virus infectivity in the alimentary canal. The authors suggested that that these advantages aided viral adaptation and resistance to high body temperatures such as fever after viral transmission to other tissues. Moreover, these advantages could also explain the low proportion of VP1-31D present in the brain after positive selection. However, the VP1-31G viruses with the dominant haplotype in the CNS exhibited high viral growth and fitness in neuronal cells which implied that VP1-31G contributed to CNS invasion and caused severe neurological diseases in patients. Speculations were that varying tissue tropism of EV-A71 among different sites of infections brought about this bottleneck for viral population with mutant spectra resulting in the adaptive VP1-31G haplotype becoming dominant in neuronal tissues. Once infection was established, VP1-31G viruses facilitated bottleneck breakthrough and invasion into the skin and CNS. Three minor haplotypes (C to E) were found to coexist in various tissues: the minor haplotypes C and D were present in the respiratory and digestive systems while the minor haplotype E was not found in the intestinal mucosa. These data indicated that EV-A71 quasispecies used the dynamic balance of different haplotype populations to cooperate, maintain population plasticity and facilitate spread to various tissues. Finally, the authors concluded that the haplotype selection could be a driving factor in viral dissemination and disease severity in humans as well as virulence in EV-A71 infected patients [55]. The consequences of limiting genomic diversity in a viral population was examined. A strain of poliovirus (G64S) with restricted genome diversity in the viral populations due to its high fidelity polymerase was found, and this strain was able to replicate as much as the wild-type poliovirus but generated less genomic diversity. As a consequence, the G64S strain was unable to adapt to adverse growth conditions. In vivo, the reduced viral diversity led to a loss in neurotropism and decreased pathogenic phenotype. Following mutagenesis whereby the quasispecies diversity was restored, it was observed that the level of virulence and neurotropism rose [56].

Complexity of the viral quasispecies also enabled the virus to spread systematically and access the CNS, perhaps due to the complementary functions of different subpopulations that facilitated adaptation to new environments. Some variants in the population might facilitate the colonization of the gut, another set of mutants might serve as immunological decoys that tricked the immune system, and yet another might facilitate crossing the blood-brain barrier. Notably it is crucial to understand the capacity of a viral clone to quickly differentiate phenotypically in vivo. Surprisingly, viruses isolated from the pancreas of mice after one passage displayed an attenuated phenotype compared to viruses from the sera of the same mice which showed similar virulence to the wild-type clone. Furthermore, viruses isolated from the spleen displayed intermediate phenotypes. To determine the genetic characteristics relevant for decreased virulence, the genomes of the entire viral population were analyzed, and the data revealed up to 29 mutations within the nonstructural proteins (12 of these led to amino acid substitutions) of the variant viruses when compared to the parental virus. These findings indicated that populations of varying virulence could co-exist at the same time in a given viral population [57].

It was proposed that the quasispecies phenomenon in a wide variety of viruses could be exploited to create stable and attenuated strains of viruses that could be used as suitable candidates in the design of LAV vaccines. Poliovirus variants (G64S, G64A, G64T, G64L and G64V) carrying the high fidelity polymerase were selected to study the effects of restricting viral quasispecies diversity on the virulence and stability of the strain compared to the wild type poliovirus. In the study, the effect of restricting population diversity on viral pathogenesis in mice was investigated. As expected, the wild-type poliovirus quickly invaded the central nervous system after intramuscular injection and caused paralysis and death within 5 days. In comparison, the high fidelity viruses were strongly attenuated with a marked delay on the onset of symptoms at the highest doses. The LD_50_ value of the wild-type poliovirus was 1.2 × 10^6^ PFU whereas the high fidelity strains achieved LD_50_ values 40–300-fold higher than the wild-type poliovirus. Furthermore, all high fidelity variants elicited poliovirus-specific neutralizing antibodies to higher levels (highest being G64S and G64A) when compared to those induced by the wild-type poliovirus. The amount of neutralizing antibodies obtained from G64L and G64V variants was also higher than the Sabin vaccine strain and similar to that of the wild-type. The authors concluded that the high fidelity replication variants were equivalent or better immunogens when compared to the Sabin 1 vaccine strain in the murine model [58]. The studies indicated that restricting the diversity of an RNA viral population by increasing the fidelity of replication of the RNA polymerase had a direct impact on pathogenesis and the ability of the virus to evade antiviral immunity. These findings are in line with the idea that RNA viruses have evolved suboptimal viral polymerase fidelity in order to allow rapid evolution and adaptation to new environments. These findings have direct practical implications for diseases caused by enteroviruses such as EV-A71. An alternative strategy to generate attenuation of RNA viruses is by directing their evolutionary trajectories towards detrimental regions in sequence space. RNA viruses such as coxsackievirus B3 and influenza A were engineered to harbour more serine and leucine codons with nonsense mutation targets. These viruses with more stop mutations were shown to suffer significant losses in viral fitness both in vitro and in vivo. They were found to be highly immunogenic and could protect mice against lethal challenge. The study showed that cornering viruses in a “risky” area of sequence space led to highly attenuated viruses that could serve as useful vaccine strains [59].

### Quasispecies through the Morphology of Plaques

However, some studies showed evidence for the presence of quasispecies within a population by looking at the morphology of plaques. Ramsingh et al. (1995) discovered that recombinant viruses led to the appearance of plaques of different sizes when grown in vitro. One recombinant coxsackievirus B4 (CV-B4) was initially grown in monkey kidney cells and yielded large plaques of about 1.0 cm while another recombinant coxsackievirus (CB4-P) yielded smaller plaques of 0.4 cm. The discovery revealed that the 5′-NTR and the VP4 genes independently influenced the plaque size. Moreover, recombinant viruses with mutations in either VP1 or VP2 coding sequences had smaller plaque phenotypes while point mutations within the VP4 sequence and substitutions in the 5′-NTR region led to bigger plaque phenotypes [60]. There is a connection between virulence and plaque sizes whereby large plaque variants expressed higher virulence when compared to the smaller plaque variants and this was used to differentiate between virulent strains of poliovirus and attenuated poliovirus vaccine strains [61].

In an attempt to determine whether more than one virus strain contributes to plaque morphology within a population, genetic and phenotypic assays were conducted with polioviruses of different genotypes. The results showed that in one particular plaque, multiple parental strains were present and this was due to the viral stock containing both single viral particles and aggregated virions. The authors concluded that aggregated virions were capable of inducing co-infection and chimeric plaque formations. Furthermore, a higher number of chimeric plaques was observed when mutation rates were high in the parental virus and indeed, and results showed that heavily mutagenized viruses led to 17% chimeric plaque formation. This study demonstrated that more than one virus could contribute to a single plaque formation and that coinfection helps in the formation of plaques in situations where the genome damage is high [62]. Therefore, quasispecies could arise from spontaneous mutations in the viral genomes within the population.

Although a direct link has been established between mutation rates, population dynamics and pathogenesis among different viruses, little is known about the effects of quasispecies and viral subpopulations of EV-A71 on pathogenicity and antigenicity. Thus far, the impact of quasispecies of EV-A71 on virulence can only be modelled and predicted based on previous studies targeting polioviruses and other RNA viruses in general. Moreover, the important question as to whether a single mutation in EV-A71 is really the root cause of fatality due to increased virulence or rather fatality is due to cooperation amongst mutants with different nucleotide mutations remained unanswered. In this regard, it would be apt to study the effects of each of these two scenarios in order to establish which of the two: cooperation between quasispecies within a viral population or specific fatal strains carrying one or more spontaneous mutations which caused increased virulence, are more likely to influence pathogenicity of EV-A71. Notably, it would provide more insight if any relationship between specific fatal strains and the phenomenon of quasispecies could be established. It would also be wise to explore the possibility of restricting the effects of viral subpopulation formation in EV-A71 in order to create a genetically more stable strain that is less likely to generate diverse variants that could escape host immunity. The outcome of such a possibility could generate an ideal candidate for a live-attenuated vaccine (LAV). Therefore, in the future, a more complete characterization of viral population and the dynamics that exist between different strains of EV-A71 during an infection could help to decipher the interplay between viral dynamics and virulence.

## 5. Conclusions

EV-A71 is an enterovirus that is currently responsible for many large-scale outbreaks of HFMD with high fatality in the Asia Pacific region. In this review, we investigated whether recombination and spontaneous mutations are the key parameters that drive the nature and diversity of a viral population. It has been revealed that recombination occurred extensively throughout the genome of EV-A71 and the data suggest that this may be a key factor in determining the heightened virulence exhibited by some strains. On the other hand, the phenomenon of quasispecies was explored. The question as to whether quasispecies are really the root cause of virulence was discussed by examining previously reported viruses such as poliovirus, rabies virus and coxsackievirus B4. There is evidence of the existence of EV-A71 quasispecies displaying broad mutant spectra with divergent mutations at the initial infection sites in the respiratory and digestive systems. After viral invasion, a major haplotype switch from VP1-31D to VP1-31G in the mutant enabled the virus to break through the selective bottleneck and invade the central nervous system in humans. Through an extensive study of recombination events, the phenomenon of quasispecies and spontaneous mutations affecting virulence could shed more light on the evolution of EV-A71 and pathogenesis. Ultimately, more complex studies looking into the behaviour of a viral population in an infection model and its connection to pathogenesis should be carried out as little is known about whether a single strain is solely responsible for fatal infection or whether it could be due to cooperation of variants carrying different mutations in the population. Providing an answer to these questions could pave the way for designing an effective vaccine against EV-A71.

## Figures and Tables

**Table 1:** Recombination events in the genome of EV-A71.

Recombinants of EV-A71	Genes of the EV-A71 Involved in Recombination	Significance of the Recombination Events	References
EV-A71 strains SZ/HK08-5 and SZ/KH08-6	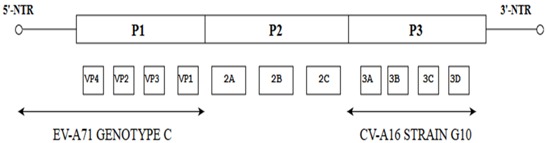	Both EV-A71 strains have >80% similarity to the EV-A71 genotype C strain (Tainan/4643/98). Both EV-A71 strains showed similarity of ≥80% to the G-10 prototype strain of CV-A16.	[29]
Recombination between species A and B human enteroviruses	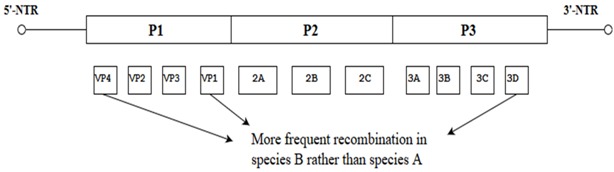	Enterovirus species B showed more recombination events between VP1 and 3D^pol^ as well as between VP1 and VP4.	[23]
Seven full-length EV-A71 C4 sequences from HFMD patients who had severe or mild diseases	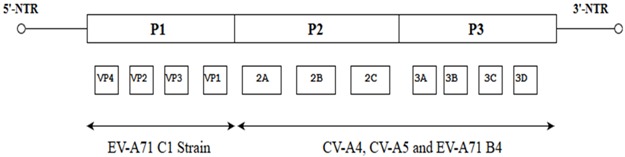	All seven strains might have originated from the same ancestor as they were found in the same cluster after phylogenetic analysis	[30]
EV-A71 subgenotypes A, B (B2, B3 and B4) and C (C2 and C4)	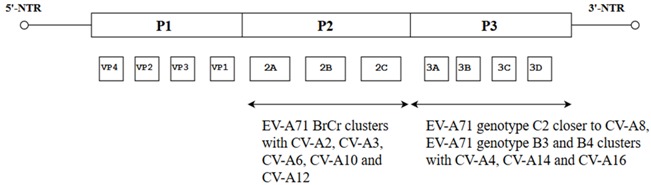	These similarity plots support the likelihood of intertypic recombination between EV-A71 and different Human Enteroviruses A (HEV-A)	[31]
EV-A71 subgenotype C2, which was observed to be circulating in Taiwan in 1998	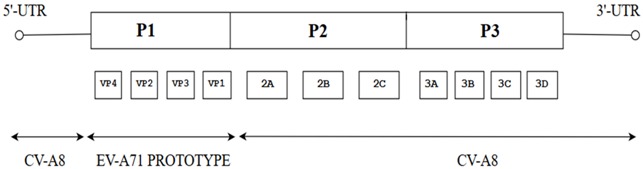	Recombination between CV-A8 and EV-A71 shows evidence of intertypic recombination.	[24]
C4 isolates, which circulated in China from 2004 to 2005	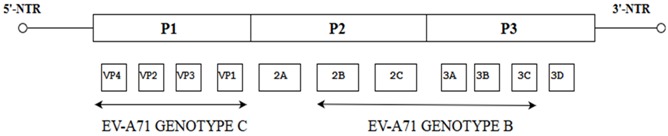	Proof of intratypic recombination was observed between EV-A71 subgenotype C and B.	[32]

Recombination was observed between EV-A71 and other enteroviruses such as CV-A16, CV-A8 as well as other subgenotypes of EV-A71.

**Table 2:** Comparison of nucleotide and amino acid identities between the prototype strain of EV-A71 (BrCr), Coxsackie A16 and wild type Poliovirus. The comparison among the different enteroviruses was reported throughout specific regions of the genome from 5′-NTR, P1, P2, P3 and 3′-NTR [13].

**Nucleotide Identity of Strain EV71/MS/7423 with Other Enteroviruses**								
	Whole Genome		5′-NTR	P1		P2	P3	3′NTR
EV-A71/BrCr	81		85	82		77	80	92
Coxsackie A16	77		86	68		82	79	79
Poliovirus	58		71	53		58	61	41
**Amino Acid Identity of Strain EV71/MS/7423 with Other Enteroviruses**								
	Whole Genome	P1	P2	P3	VP1	VP2	VP3	VP4
EV-A71/BrCr	95	97	94	94	93	99	99	100
Coxsackie A16	89	79	95	95	71	84	84	78
Poliovirus	55	46	59	62	36	55	45	58

**Table 3:** Spontaneous mutations in different EV-A71 subgenotypes (BrCr, B1-B5, C1-C5).

Mutant Strains of EV-A71	Position of Amino Acid(s) on the EV-A71 Genomes of Mutants	Significance of the Mutations in the EV-A71 Genome	References
Analysis of EV-A71 subgenotype C4 showed changes in the 5′-NTR and the VP1.	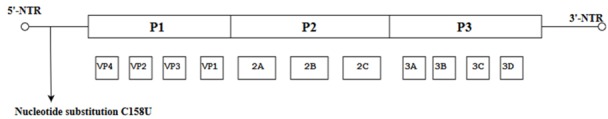	When the nucleotide cytosine was substituted with uridine at position 158, the conformation of the RNA secondary structure of stem loop II in the 5′-NTR changed, leading to a decrease in viral translation and virulence in mice.	[38]
Nucleotide and amino acid changes in neuro-virulent strains of EV-A71 subgenotype C4a.	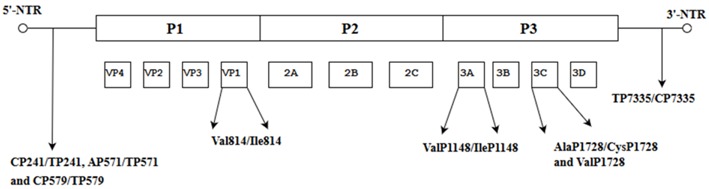	These amino acids are potential molecular determinants of virulence. Variations in the secondary structure of the 5′-NTR at three positions (C^P241^/T^P241^, A^P571^/T^P571^ and C^P579^/T^P579^) and one position in the 3′-NTR(T^P7335^/C^P7335^) might confer fatality.	[39]
Comparisons of EV-A71 across different genotypes (BrCr, B1-B5 and C1-C5)	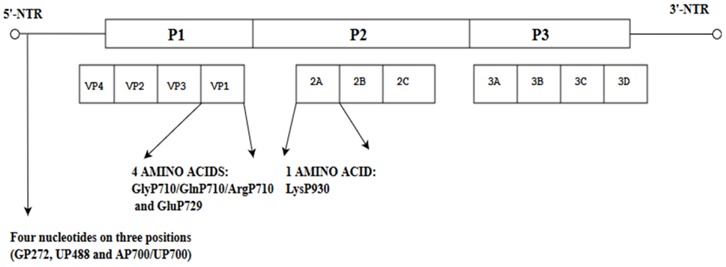	These amino acid residues might be associated with the EV-A71 virulentphenotype.	[40]
Changes in VP1 sequences of EV-A71 subgenotype C4 causing severe HFMD	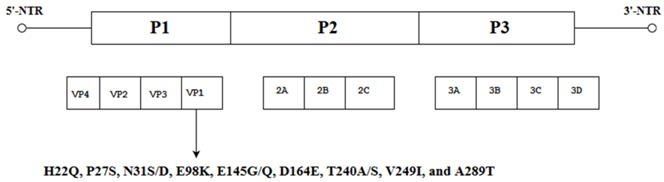	E145Q/G interacts with residues of the PSGL-1 N-terminus and acts as a molecular switch to modulate binding to the cell receptor by controlling the exposure of the amino acid (VP1-244K) on the VP1 surface.	[41,42]
Analysis of EV-A71 subgenotype C4 showed changes in the 5′-NTR and the VP1	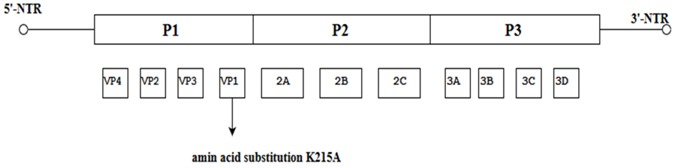	K215A located at the VP1 GH loop increased the thermal stability of the virus.	[44]
Roles of K244E and H37R were investigated by reverse engineering in the EV71-B2 isolate, MS/7423/87	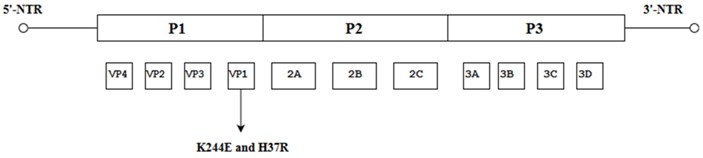	It was postulated that H37E and K244E interactions were important for replication in primate cells but K244E alone was able to confer the ability of the virus to replicate alone in a murine model	[45]
Role of K^216^→R, G^145^→E and K^129^→I in the mouse-adapted strain of EV-A71 26M/AUS/4/99	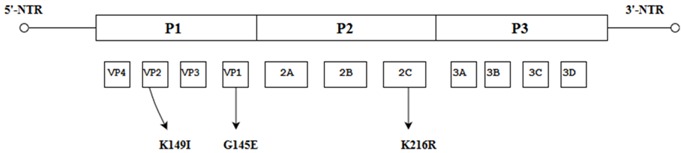	G^145^→E mutation was solely responsible for an increase in virulence in mice whilst K^129^→I led to an improved growth of the strain in vitro but did not lead to increased virulence in mice	[48]
Analysis of the genomes of six EV-A71 strains of subgenotype C4a identified the only change of amino acid Asn 1617 in the 3C gene	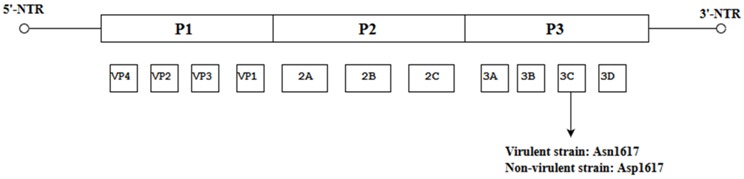	This specific amino acid led to conformational change at the active centre of the 3C proteinase (3C^pro^) and this could be a potential molecular determinant for the EV-A71.	[49]

Analysis of different EV-A71 strains showing the position of the spontaneous mutations present in the genomes of EV-A71 and their significant impact on virulence.
